# How to manage non-response using stimulants?

**DOI:** 10.1192/j.eurpsy.2025.225

**Published:** 2025-08-26

**Authors:** A. Philipsen

**Affiliations:** Privatpsykiaterne, Sorø, Denmark

## Abstract

**Abstract: Introduction/background:**

Psychiatrist working with ADHD-patients since 2007.

**Method:**

I have diagnosed and treated more than a 1000 patients with ADHD. I have always, when it has been possible, given one medication at a time to be able to evaluate how it works, and what gives side effects.

**Results:**

When stimulants suddenly don’t work anymore, I have searched for physical and psychological problems that could explain it. By solving these problems, the stimulants work again.

**Conclusion:**

By working this way, we have noticed that patients have a high compliance, and there are a lot less cases of relapse with ADHD symptoms.

**Disclosure of Interest:**

None Declared

